# Reduced Abundance and Subverted Functions of Proteins in Prion-Like Diseases: Gained Functions Fascinate but Lost Functions Affect Aetiology

**DOI:** 10.3390/ijms18102223

**Published:** 2017-10-24

**Authors:** W. Ted Allison, Michèle G. DuVal, Kim Nguyen-Phuoc, Patricia L. A. Leighton

**Affiliations:** 1 Centre for Prions & Protein Folding Disease, University of Alberta, Edmonton, AB T6G 2M8, Canada; thienkim@ualberta.ca (K.N.-P.); pleighto@ualberta.ca (P.L.A.L.); 2 Department of Biological Sciences, University of Alberta, Edmonton, AB T6G 2E9, Canada; mgduval@ualberta.ca; 3 Department of Medical Genetics, University of Alberta, Edmonton, AB T6G 2M8, Canada

**Keywords:** protein homeostasis, prion-like disease, amyotrophic lateral sclerosis, functional amyloid, scrapie, premelanosome protein (PMEL17), strains, SOD1

## Abstract

Prions have served as pathfinders that reveal many aspects of proteostasis in neurons. The recent realization that several prominent neurodegenerative diseases spread via a prion-like mechanism illuminates new possibilities for diagnostics and therapeutics. Thus, key proteins in Alzheimer Disease and Amyotrophic lateral sclerosis (ALS), including amyloid-β precursor protein, Tau and superoxide dismutase 1 (SOD1), spread to adjacent cells in their misfolded aggregated forms and exhibit template-directed misfolding to induce further misfolding, disruptions to proteostasis and toxicity. Here we invert this comparison to ask what these prion-like diseases can teach us about the broad prion disease class, especially regarding the loss of these key proteins’ function(s) as they misfold and aggregate. We also consider whether functional amyloids might reveal a role for subverted protein function in neurodegenerative disease. Our synthesis identifies SOD1 as an exemplar of protein functions being lost during prion-like protein misfolding, because SOD1 is inherently unstable and loses function in its misfolded disease-associated form. This has under-appreciated parallels amongst the canonical prion diseases, wherein the normally folded prion protein, PrP^C^, is reduced in abundance in fatal familial insomnia patients and during the preclinical phase in animal models, apparently via proteostatic mechanisms. Thus while template-directed misfolding and infectious properties represent gain-of-function that fascinates proteostasis researchers and defines (is required for) the prion(-like) diseases, loss and subversion of the functions attributed to hallmark proteins in neurodegenerative disease needs to be integrated into design towards effective therapeutics. We propose experiments to uniquely test these ideas.

## 1. Introduction

**Figure 1:**
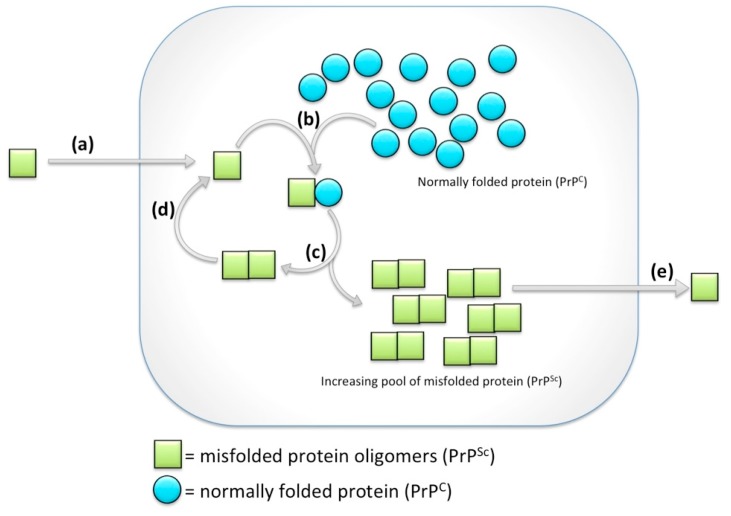
A highly simplified schematic of template directed misfolding in prion-like diseases. Temporal events that make up a cycle of template-directed misfolding: (**a**) A molecule (or oligomer or aggregate) of misfolded prion (PrP^Sc^, represented by green square) arrives to a naïve tissue or cell. This spreading to new locations initiates proteostatic disruption and degenerative events in otherwise healthy cells; (**b**) Normally folded prion protein (PrP^C^, i.e., “cellular” form represented by blue circles) is recruited to interact with PrP^Sc^; (**c**) Thus PrP^Sc^ accumulates in the new cell, with two consequences; (**d**) The PrP^Sc^ fragments (e.g., oligomers or fibrils) break apart and recruit more PrP^C^ into the misfolding cycle; (**e**) PrP^Sc^ continues to spread to new cells and tissues. All the events in this model represent functions gained upon protein misfolding, whereas our synthesis asks if disease etiology ought to also consider functions that are lost following protein misfolding.

Prion diseases are incurable neurodegenerative diseases of people, livestock and wildlife that devastate individuals and families, socioeconomics of agricultural sectors, and the ecology surrounding cervids (deer, moose, caribou/reindeer, etc.). Prion diseases are impactful despite being relatively rare. During prion disease the normally folded cellular prion protein (PrP^C^) misfolds (denoted “scrapie” form, PrP^Sc^) (Figure 1) and gains functions such as toxicity and infectivity. When PrP^C^ misfolds it also loses functions, though the functions of PrP^C^ are not yet fully appreciated (see below), and we argue here that loss and subversion of the functions attributed to PrP^C^ in neurodegenerative disease needs to be integrated into design towards effective therapeutics.

Prion disease concepts have for several decades informed key mechanisms in neurology especially with respect to protein homeostasis [1]. Most recently, the classical prion diseases have inspired a new conception of several progressive neurodegenerative diseases that are increasingly common in the aging first world population: thus prominent neurodegenerative diseases are now broadly agreed to be “prion-like”, including Alzheimer, Parkinson, Huntington Diseases and Amyotrophic Lateral Sclerosis [2,3,4,5,6,7,8,9,10]. This recent insight continues to inform new diagnostic and therapeutic options. Thus prions have taught researchers much about a broader class of neurodegenerative diseases; Here we seek reciprocity and suggest that comparison amongst the prion-like diseases ought to be mined to identify commonalities and distinctions that might inform the canonical prion diseases.

Applying this framework has led us to encourage an increased level of attention upon protein loss of function (LOF) during protein misfolding/aggregation/amyloidosis as a causative contributor to disease progression, including LOF in the keystone disease proteins such as the prion protein itself [7]. This is meant to encourage attention on misfolding that complements the current major focus on misfolding leading to a gain of function (GOF), i.e., a gain of toxic function that produces disease. Thus GOF is required in the definition of what makes a prion disease, however GOF may not be the sole driver of disease aetiology. Here we update this synthesis by deeper consideration of the protein SOD1 (superoxide dismutase 1) as an exemplar of the concept. We expand upon this by asking if the proteostatic mechanisms revealed by SOD1 are occurring in other prion-like diseases (i.e., following SOD1 misfolding, its function is reduced—see below). We then uniquely ask if lessons regarding loss-of-function during protein aggregation can be derived from considering functional amyloids that are critical to normal physiology. We end by suggesting experiments to assess the ideas emerging from our synthesis. First we will begin by reviewing what characters are shared by neurodegenerative diseases that newly classify them as being “prion-like”.

## 2. Prion-Like Diseases

Prions, i.e., misfolded toxic protein confomers, spread between tissues and cells where they have a propensity to induce further misfolding [11]. Within this naïve tissue, the newly arrived misfolded prion interacts with properly folded physiological protein confomers and causes them to misfold in a process of template-directed misfolding (Figure 1).

Prion diseases are unique in possessing three modes of initiation: acquired, genetic and sporadic. Thus the appearance of misfolded protein species that instigates prion diseases (e.g., Figure 1 step (a)) can occur by exposure to infected material (e.g., via oral routes, iatrogenic exposure via surgical instruments or tissue grafts, etc.). Further, genetics can drive the misfolding of the instigating protein by changing amino acids that increase propensity for protein misfolding. On the other hand, when explanations such as genetics and infection are not obvious the disease initiation is described as “sporadic”; this indeed may often be a sporadic misfolding of the protein in an otherwise perfectly healthy uninfected cell, but may also be due to unappreciated causality abased in genetics or infection.

The list of diseases that are now recognized as being prion-like has rapidly expanded over the recent years, now including prominent neuropathologies such as ALS, Parkinson, Huntington, and Alzheimer Diseases (via both Aβ and Tau) [2,3,4,5,6,7,8,9,10]. The quintessential proofs entail infecting naïve animals via delivery of brain homogenate from diseased animals, or delivery of misfolded protein seeds alone, and assessing if they nucleate disease.

## 3. Protein Functions are Lost, Reduced and Subverted upon Protein Misfolding: Yet Gained Functions Fascinate the Prion Biologist

**Figure 2:**
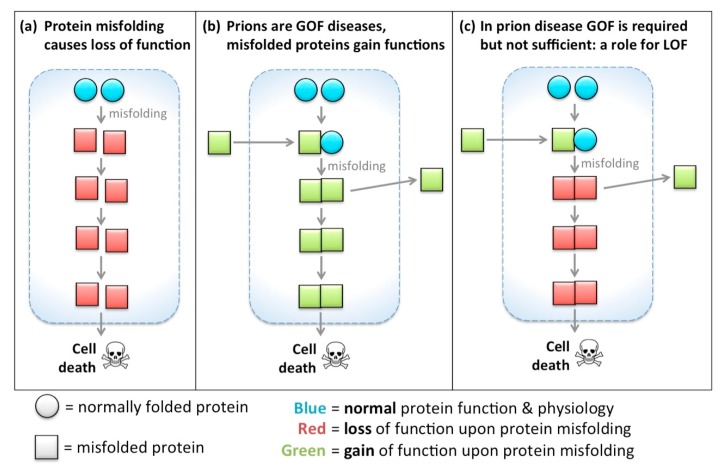
Gain-of-function concepts dominate the prion field, but a protein’s functions are also lost when it is misfolded. Simplified schematic to represent the hypotheses regarding proteins that exhibit gain-of-function (gain of function (GOF): labelled “green for gained/go”) when they misfold/aggregate. In other instances, protein misfolding leads to a reduction, subversion or loss of function (loss of function (LOF): labelled “red for reduced/stop”). (**a**) Upon misfolding, proteins lose their function. In most fields of biomedicine, including much of neurology, protein misfolding is associated with LOF, and LOF is invoked to explain disease etiology; (**b**) Misfolded prion-like protein arrives to a new cell or location and induces misfolding of resident normal protein (e.g., see Figure 1). Prions are a remarkable class of diseases rooted in protein misfolding, wherein the misfolded protein gains functions (including the ability to induce template misfolding of other proteins, and to be infectious when applied to naïve cells or individuals). GOF is required in prion diseases. Because GOF defines prion diseases and offers key opportunities to intervene in disease course, prion biology has a tendency to consider prion diseases as being almost exclusively a protein GOF disease. Thus this schema shows no role for protein LOF; (**c**) Our key hypothesis is a re-envisioning of prion-like diseases, wherein LOF dominates etiology, as in most diseases wherein proteins misfold. Even in this extreme view GOF is required—the distinction to panel b is that GOF is not sufficient, and LOF is required for disease outcomes.

In most diseases, protein misfolding (due to mutation or otherwise) during disease is intuitively associated with the proteins losing their functionality as a key step in aetiology. Examples include prevalent diseases such as cystic fibrosis [12,13], cancer [14,15,16,17,18,19,20], diabetes [21], myopathies [22,23] and several neurodegenerations (e.g., retinitis pigmentosa, age-related macular degeneration) [24]. Indeed the unfolded protein response is thought to be a key system in managing cellular health following injuries and during disease [20]. In most fields of biomedicine, when protein misfolding is correlated with disease course, or is predicted based on modelling a mutation’s effect, LOF is intuited to occur. Neural/cell dysfunction and degeneration follow, if not mitigated through various mechanisms such as autophagy or the unfolded protein response, and are often invoked as sufficient to explain disease etiology (Figure 2a).

From an outsider’s perspective, then, one may ask why in prion biology is the emphasis so predominantly on functions gained during protein misfolding? The answer is rooted in the definition of what makes a prion, and what fascinates about the field. Protein misfolding in prions produces its hallmark function of being infective. This infectivity is the driver of public health fear, ecological disaster, socioeconomic collapse, and iatrogenic transmission. Thus when prions misfold they gain several functions: (i) the propensity to initiate template-directed misfolding; (ii) infectivity; (iii) stability; and (iv) toxicity.

Several of these gained functions explain the high level of interest for the realization that prominent neurodegenerative diseases progress via prion-like mechanisms. These gained functions offer significant new modalities that inspire both diagnostic and therapeutic innovations. Several innovations from prion biology might then be impactful upon other diseases. The gained functions of template-directed misfolding have provided the prion biologist new diagnostic tools that exploit seeded/template misfolding to detect small amounts of prions in complex tissues. These assays include PMCA and RT-QuIC (protein misfolding cyclic amplification and real-time quaking induced conversion) that are faster and less expensive than the traditional testing of materials via animal assays (e.g., inoculation into mice or hamsters) [25,26,27]. The success of these has inspired development of equivalent assays in other prion-like diseases [28,29,30,31]. Separately, the gained function of infectivity, including the spreading of neurodegeneration within an individual, is a critical aspect of prion biology that uniquely holds promise to be targetted by new therapeutics. Small molecules or antibodies that are able to preclude template-directed misfolding and/or block spread of aggregates to naïve tissues provide a novel arena for rational design of therapeutics.

The gained character of protein stability is also critical to most every aspect of prion biology’s impact on human and animal health and ecology. Operationally it is the stability of the prion that largely enables its infectivity. Stable PrP^Sc^ is challenging to disinfect from surgical instruments and stable in tissue transplants, leading to iatrogenic infection. Stable PrP^Sc^ is resident in the soil of farm plots and forest floors long after sick livestock or cervids have left the area. Stable PrP^Sc^ is not cleared from the cell, challenging the proteostasis field to design new interventions towards health. The stability of PrP^Sc^ gained upon its misfolding is thus a key driver of challenges in reducing disease burden in patients, agriculture and ecology.

Upon misfolding prions also gain the property of increased toxicity, and this has of course inspired many investigations regarding mechanisms and interventions to alleviate toxicity. While it is often assumed that the misfolded/aggregated/amyloid forms of prion-like proteins are toxic, the mechanisms and proofs remain diffuse. It may be that misfolded/aggregated proteins accumulate sufficiently to overwhelm the cell. The balance of clearance vs. accumulation of misfolded proteins, and understanding the factors that affect such kinetics, have been a cornerstone of neurodegeneration research [32,33]. Less is known, however, as to what molecular triggers form pathways between protein aggregations/inclusions to produce neuron death. Certainly these mechanisms can include unfolded protein response regulating cells towards autophagy and apoptosis. Importantly, though, for several diseases it is increasingly recognized that dysfunction of synapses is a strong correlate to clinical disease presentation [34], and the accumulation of protein aggregates and inclusions is a later process and less well correlated [35]. In the extreme version of this point it can be argued that infamous hallmarks of protein accumulation, such as plaques rich in Aβ, have quite a poor correlation to disease presentation; indeed Aβ plaques can be richly abundant in the brains of healthy individuals [36]. Thus plaques in Alzheimer Disease (AD) could be interpreted as protective, serving as sinks of molecules (e.g., Amyloid β Oligomers, or AβO) that would be more toxic if they were free floating amongst normal synaptic physiology. In Sum, one key gain-of-function in prion-like diseases is represented by toxic protein aggregates, and this is typically viewed as sufficient to kill neurons but the mechanisms remain to be fully elucidated. Defects in neuron physiology and loss of synaptic connectivity are emerging as the strongest predictors of clinical progression, and these events might well be driven by lost (reduced/subverted/ill-deployed) protein function rather than via proteins gaining new functions in their prion-like conformations. Regardless of important instances where misfolded proteins might not be as toxic as once was assumed, toxicity remains as a topic of strong interest amongst prion biologists.

Gained functions in prion biology also fascinate at the level of evolution. Strains in misfolded protein likely represent different conformational space, and several confomers co-exist as per the “cloud hypothesis” [37,38]. In a pseudo-Darwinian competition for resources and self-propogation: Efficient strains dominate and indeed might be first to push the boundary of the “population’s” footprint, such that efficient strains spread first to naïve territory (tissues). From the perspective of therapeutics, such strain competition is expected to be very impactful on the efficacy of treatments that affect only a subset of strains. Thus if a small molecule inhibitor or antibody against a disease-specific epitope were successful in removing only some subset of strains, the remaining strains might be able to emerge and accelerate, thereby thwarting treatment efficacy [39].

In the broader sense, prions also challenge the Central Dogma of Biology that information is carried in DNA and RNA [40,41,42]. Prions reveal that different biological outcomes can be encoded in protein conformations (rather than in protein primary sequence).

In Sum, gained functions fascinate the prion biologist for good reason. Indeed gained functions are what define a prion and distinguish it from other protein systems. Gained functions of prions allow optimism for new diagnostics and therapeutics in common devastating neurologies, set challenges to be met in reducing disease burden, and inspire wholly novel questions in evolutionary biology. GOF will continue to dominate the prion field, as it should. In this synthesis we bring together elements that encourage the field to more frequently consider a complementary suite of outcomes centered in LOF, and hope to convince that integrating LOF may be a key aspect of several key prion biology challenges.

Although a focus on gain-of-function has fascinated, and prions have been pathfinders for understanding, it is useful here to outline two complementary hypotheses (see also Figure 2c):

1. H_0_: Gain of function is required in prion disease, but not sufficient.
2. H_0_: Loss of function is not sufficient in prion disease, but is required for progression.

Thus we wish to emphasize our speculation that while gain of function is required in prion disease, and is very reasonably the center of much attention in prion biology, GOF is probably not sufficient for disease and opportunities to understand disease course and interventions are thus being missed.

As a mirror of this, Loss of Function is unambiguously not sufficient in prion disease, but we compile evidence favouring the hypothesis that loss of function(s) in proteins like PrP^C^ are likely required for disease progression. The conclusion that LOF is not sufficient seems nearly unassailable because *Prnp* knockout mice do not develop prion disease (However it is intriguing that acute loss of PrP^C^ homologs in zebrafish induce neurodevelopmental phenotypes [43]). Others have recognized the potential role for LOF in prion disease [44]. If, as we assert here, loss of function is a key part of etiology towards neuron death and dementia, then this has significance in two applied areas: (1) preventing loss of function may be a therapeutic strategy; (2) promising therapeutic strategies that seek to reduce PrP^C^ abundance in order to prevent disease progression may be ill-fated, or perhaps can be improved upon by considering strategies to ameliorate disease-promoting effects of their loss of function.

The matter is complicated by the fact that abundance of PrP^C^ is positively correlated with disease progression—PrP^C^ is required for disease and adding more of it to the system would accelerate progression. Understanding the physiological roles of PrP^C^ is therefore fundamental to developing effective treatments—what are the function(s) of PrP^C^ in healthy brains, and how can they be modulated to be at near-normal levels when PrP^C^ function(s) are lost, mistimed or subverted?

Here we use the term protein loss-of-function (LOF) as a concise term to capture a broader and more nuanced concept. It is useful to consider that LOF might instead be a reduction of function, or may represent a loss of some of the diverse functions attributed to each of the proteins under consideration (see below). The functions lost upon misfolding may differ based on organelle chemistry or the presence and activation state of protein interactors. Thus LOF may be different in different tissues, different cells or cell compartments. Such complexity is imposing but encourages that a rich suite of molecular mechanisms awaits discovery. Indeed we argue below that such mechanisms associated with the varieties of LOF will be useful to improve or imagine therapeutics in prion-like disease.

## 4. Physiological Roles for PrP^C^: Lost Functions Resemble Disease Etiology

**Figure 3:**
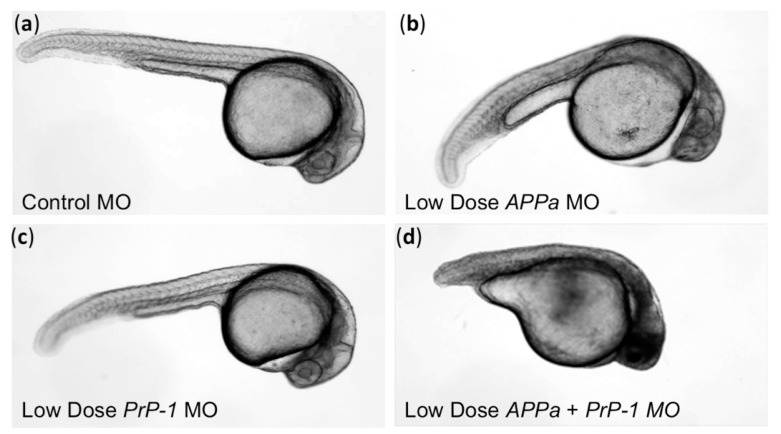
Loss of prion protein (PrP) induces neurodevelopmental phenotypes in zebrafish. Some of the functions revealed by morpholino (MO) knockdown of prion protein in zebrafish may overlap with the functions of Amyloid β Precursor Protein (APP), as suggested by the synergistic phenotype revealed when they are disrupted together. (**a**) Delivery of control morpholino (MO) reagents show typical zebrafish development; (**b**,**c**) Small reductions of either APP or PrP leads to subtle phenotypes and some CNS cell death (dark areas in head), whereas delivery of high dose APP MO induces more dramatic phenotypes; (**d**) Combining the low dose knockdown of APP *and* PrP leads to dramatic developmental defects. Human and mouse homologs of APP and PrP are able to rescue the phenotype in (**d**) when delivered to developing zebrafish. Numerous control experiments support the specificity of these reagents [43]. Images modified from [43].

Cellular prion protein (PrP^C^) is an abundant GPI-anchored protein present at cell membranes [45]. Prion protein is robustly expressed in the CNS and present in most tissues [7,46]. It is highly conserved throughout mammals, and sufficiently conserved between fish and mammals that (i) mammalian orthologs can rescue phenotypes that occur when PrP^C^ homologs are reduced in zebrafish (Figure 3) [43]; (ii) zebrafish PrP^C^ is properly processed regarding post-translational modifications when expressed in mammalian cells [47]. PrP^C^ is highly conserved within mammals despite being a template for progression of an invariably fatal disease. Thus it seems that the function of PrP^C^ must be important. Loss, reduction or subversion of some PrP^C^ functions may be causal in disease etiology.

**Figure 4:**
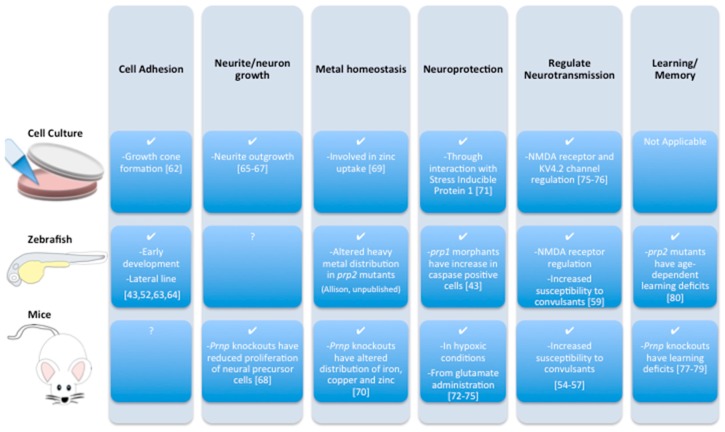
Cellular prion protein (PrP^C^) has several functions attributed to it that have been derived from characterizing cells, fish or mice lacking PrP^C^.

Despite years of research related to the role of PrP^C^ in prion disease, PrP^C^’s raison d’être is not well-established [7,46,48]. While many putative functions of PrP^C^ have been uncovered, early efforts to identify functions critical to organism health were thwarted by the lack of overt phenotypes in *Prnp* knockout mice [49,50,51,52]. PrP^C^ has several functions attributed to it, derived from characterizing cells, fish or mice lacking PrP^C^ (Figure 4). Some of these functions map onto disease symptoms observed in patients. For example, seizures are a feature of a small number of prion disease cases [53] and PrP^C^’s role in protection against seizures is demonstrated in *Prnp* knockout mice [54,55,56,57,58]. Analysis of mutant zebrafish has confirmed that PrP^C^ regulates NMDA receptor kinetics and regulates neural activity towards protecting from seizures, confirming similar phenotypes in mice and suggesting that PrP^C^ had ancient functions regulating neural activity [59]. Prion mutant mice exhibit disrupted circadian rhythms [60] and the parallels of this to patients suffering Fatal Familial Insomnia are discussed below (see end of Section 6). Further, roles of PrP^C^ in neuroprotection and learning are interesting both in light of cognitive deficits observed in prion disease patients [61] and with respect to PrP^C^’s recent connection with Alzheimer’s disease, detailed below. Thus the function of PrP^C^ is not well-established, though some functions (learning, neuroprotection) are very intriguing observations in the context of dementia but do not yet have a molecular mechanism [43,52,54,55,56,57,59,62,63,64,65,66,67,68,69,70,71,72,73,74,75,76,77,78,79,80].

**Figure 5:**
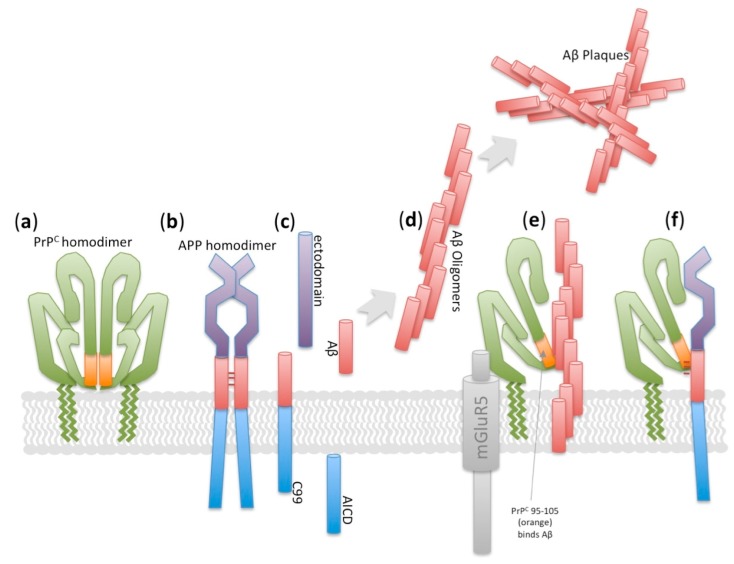
Diverse roles for Cellular prion protein (PrP^C^) in Alzheimer Disease physiology, supporting that loss of PrP^C^ might impact upon neurodegeneration. PrP^C^ interacts both with amyloid β precursor protein (APP) and with its catabolite amyloid β oligomers. (**a**) PrP^C^ is a GPI-anchored protein that can dimerize with itself, and is present in the same cellular compartments as APP; (**b**,**c**) Homodimerization of APP impacts upon its cleavage by β- and γ-secretases into amyloid β (Aβ) and APP intracellular domain (AICD); (**d**) Aβ oligomerizes and forms plaques; (**e**) PrP^C^ is a high-affinity receptor for Aβ oligomers and mediates signals to the intracellular compartment via mGluR5; (**f**) Biochemical and genetic data support that PrP^C^ and APP also interact.

PrP^C^ is now recognized to be a high-affinity receptor for amyloid β oligomers (AβO) [81,82], which appears to be the most toxic/impactful species of amyloid β in AD (Figure 5). PrP^C^ signals to the intracellular compartment via mGluR5 to the kinase Fyn, and this provides one linkage to Tau hyperphosphorylation that might account for AD progression [83,84]. PrP^C^ also appears to have an important interaction with the parent protein from which AβO are metabolized, Amyloid β Precursor Protein (APP) [43] (Figure 5). Co-immunoprecipitation and in vivo interactome data are consistent with a physical interaction of these proteins [43,85,86,87], and zebrafish have been used to support their interactions at the genetic level including support for mammalian proteins being able to replace zebrafish proteins during the interactions [43] (Figure 3). PrP^C^ is reduced in Alzheimer patients [88]. On the other hand, these changes in AD patient brains were not reflected in mice because knockout of *Prnp* had no observed effect in one transgenic mouse model of AD [89], though the mouse model only partially represents the cellular outcomes observed in AD. In sum, mechanistic roles for PrP^C^ in the pathways leading to AD continue to be revealed, including via interactions with both Aβ and APP and especially at the synapse. Thus PrP^C^ LOF is associated with neurodegeneration and dementia; that this association occurs in the absence of PrP^C^ misfolding and in the absence of GOF mechanisms is broadly consistent with our framework that GOF is not the only route through which PrP^C^ can influence dementia.

## 5. Physiological Roles for SOD1: Functions Whose Loss Can Rationally Explain Etiology

SOD1 is most well-known for converting the superoxide anion O_2_ into O_2_^−^ or H_2_O_2_, thereby clearing free radical byproducts that would cause oxidative stress. But the oxidizing ability of SOD1 is utilized in other cellular processes, including activating proteins and producing peroxide to regulate signal transduction, gene expression, proliferation, differentiation, and cell death [90,91]. SOD1 is ubiquitously expressed, acting in the cytoplasm and intermembrane region of the mitochondria [92]; in cells with high energy demand including motor neurons, SOD1 activity at the mitochondria is especially critical to prevent oxidative stress and associated damage, including at the neuromuscular junction (NMJ). Extracellular SOD1 may also contribute to neuroprotection by raising intracellular calcium, possibly by activating the phospholipase C/PKC pathways [92,93], as well as interacting with neighbouring glia and muscle tissue.

**Figure 6:**
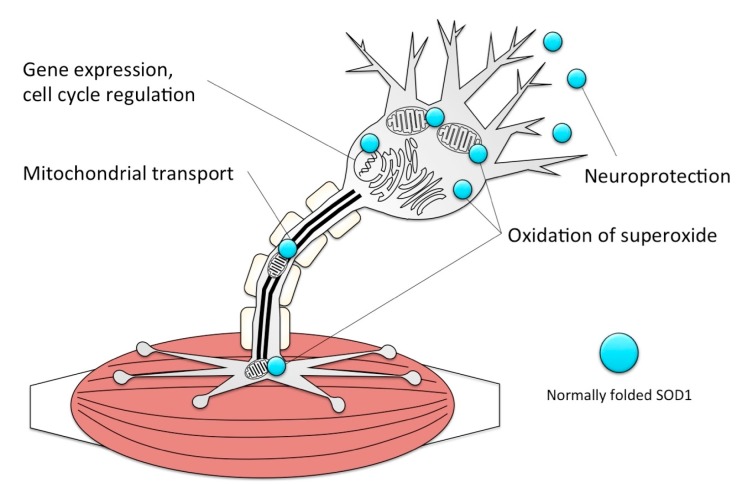
Functions of superoxide dismutase 1 (SOD1) in motoneurons. SOD1 is located in the cytoplasm, synapse and intermembrane space of mitochondria. SOD1’s role is to oxidize free radicals, notably superoxide O_2_^−^, thereby mitigating oxidative stress. It is also secreted and thought to have neuroprotective effects extracellularly. SOD1 can change the activation states of other proteins, thereby regulating gene expression, proliferation, and differentiation. Loss of function studies have shown that SOD1 also participates in axonal trafficking of mitochondria to the synapse.

SOD1 is one of the most common genes implicated in ALS, accounting for approximately 20% of all familial ALS (fALS) cases, and over 180 mutations in SOD1 have been identified in patients to date [94]. SOD1 function is critical to motoneurons, and especially to the neuromuscular junctions, where its role is to oxidize free radicals, notably superoxide O_2_^−^, thereby mitigating oxidative stress (Figure 6).

SOD1 misfolding can spread in a prion-like manner. Evidence for and against prion-like mechanisms has been extensively reviewed by other colleagues [95], including observed seeded propagation in vitro, conformation changes [95] detected using antibodies and altered protease activity, and evidence that conversion hinges on a sequence specificity characteristic of a species barrier [95,96,97]. Whether this propagation is accomplished via template directed misfolding or nucleated polymerization (or perhaps both) is still to be elucidated [95,97,98,99].

The spread of SOD1 aggregates in cell culture models strongly points to a prion-like behaviour, and its spread in vivo is not inconceivable; misfolded proteins have been demonstrated to spread via macropinocytosis, exosomes, synaptic vesicles, or as dying cells release their contents [95,97]. Neurons, astrocytes, and glia are known to exchange vesicle contents, and SOD1, a cytoplasmic protein, is commonly found in these. The uptake of mutant SOD1 in cultured neuronal cells causes aggregation of the endogenous (mutant) SOD1, which persists over cell divisions after the original seeds dissipate [97]. There is evidence for mutant misfolded SOD1 inducing wildtype, normally-folded SOD1 to misfold, and misfolded wildtype SOD1 can also act as a seed in cultured cells, as aggregates of misfolded SOD1 spread from cell to cell [100]. Prion-like propagation has been confirmed in vivo via seeding in transgenic mice that express large amounts of mutant SOD1, which is currently the closest evidence of protein propagation for misfolded SOD1 [96,101]. It should also be considered that delivery of misfolded SOD1 might also act to accelerate disease (rather than or in addition to initiating misfolding). True prion-like infectivity (i.e., “infection” of a naïve host) has not been observed.

Nonetheless, for the purposes of the logic herein, it matters little whether or not SOD1 meets the most strident definitions of being prion-like i.e., whether or not misfolded SOD1 can induce disease in a naïve healthy individual. What is clear from the literature is that misfolded SOD1 can induce template-directed misfolding of SOD1 in adjacent cells and tissues. This property is sufficient to rationalize all the mechanisms we discuss in this manuscript, because all the imagined processes are occurring at the level of cells and tissues, not between individuals. In Sum, for the purposes of this work we are likely safe to assume SOD1 qualifies as being prion-like; in other contexts, this assumption may be found wanting until more in vivo trans-individual inoculations have been assessed.

SOD1 mutations do not occur in all cases of familial ALS, and fALS does not account for the majority of cases. If SOD1 is a primary agent of disease, the presence of misfolded wildtype SOD1 in sporadic ALS would further implicate a prion-like mechanism of spread. SOD1 inclusions have been detected in tissues of many ALS patients, including sALS with no SOD1 mutations, but whether misfolded SOD1 occurs in all cases of sALS is yet to be resolved [102,103]. Despite the flourish of important new genetic loci with much explanatory power regarding ALS etiology and progression, SOD1 thus remains as a hallmark feature and its misfolding is believed to play a role in many (if not most) cases of ALS.

## 6. Loss of SOD1 Following Misfolding: Similarities in Prion but Not Other Prion-Like Diseases?

**Figure 7:**
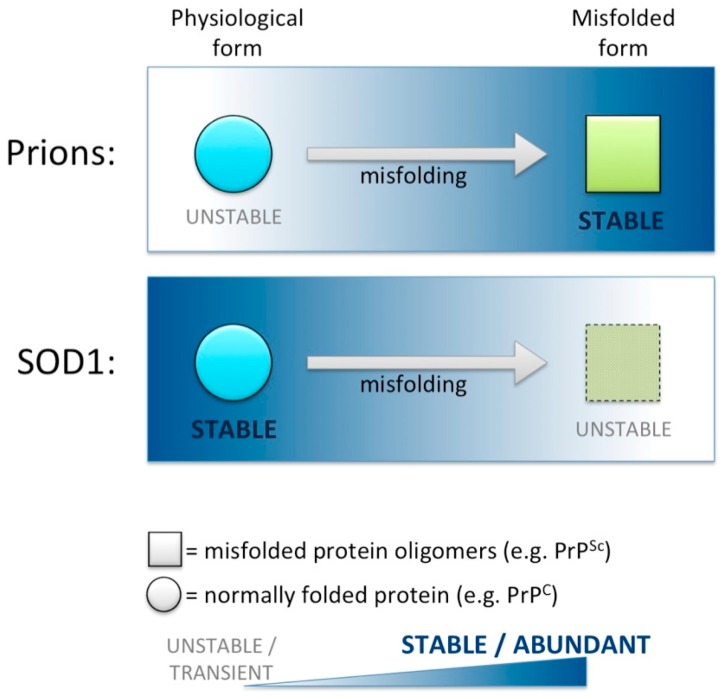
When comparing their physiological and misfolded forms, the stability profile of SOD1 is inverted compared to Prion Protein. Top: Properly folded Prion Protein (PrP^C^, the normal physiological cellular form) has a typical protein stability and proteostatic clearance kinetics, whereas the misfolded scrapie form (PrP^Sc^) is infamously stable. The extreme stability of misfolded prion PrP^Sc^ creates important challenges in clearing BSE (bovine spongiform encephalopathy) from contaminated agricultural fields, clearing CWD (Chronic Wasting Disease) from deer habitats/ecology, and disinfecting surgical instruments. Bottom: SOD1 is now appreciated as being prion-like, and we note it as an exemplar of protein functions being lost during etiology, in part because its proteostatic profile is opposite that of prion protein. Physiological normally folded SOD1 is extremely stable, such that it infamously can be recovered in active form from ancient mummified remains. Misfolded SOD1 is remarkably unstable such that it is reduced in function, in sharp contradistinction to PrP^Sc^ accumulation.

ALS is an especially informative case study in the analysis of the prion-like disease class with respect to a putative role for protein LOF. In particular, misfolded SOD1 is a toxic GOF prion-like entity (see above), but exhibits a proteostatic profile that is inverted compared to classical prion disease (Figure 7).

Properly folded PrP^C^ has a roughly typical protein stability and proteostatic clearance kinetics, whereas the misfolded Scrapie form (PrP^Sc^) is infamously stable. The extreme stability of PrP^Sc^ creates important challenges in clearing BSE (bovine spongiform encephalopathy) from contaminated agricultural fields, clearing CWD (chronic wasting disease) from deer habitats/ecology, and disinfecting surgical instruments. This is in sharp contradistinction to the pattern of SOD1 protein stability: Physiological normally folded SOD1 is extremely stable, such that it infamously can be recovered from ancient mummified Egyptian remains [104,105]. Similarly, SOD1 in its physiological form is stable in biochemical conditions (SDS, Urea) that misfold most protein [106]. Misfolded SOD1 is notably unstable compared to its physiological form, and therefore might be reduced in abundance, likely being cleared by proteostatic mechanisms, in sharp contradistinction to PrP^Sc^ accumulation (Figure 7). Further, misfolded SOD1 has substantially reduced enzymatic activity [107], as described below.

**Figure 8:**
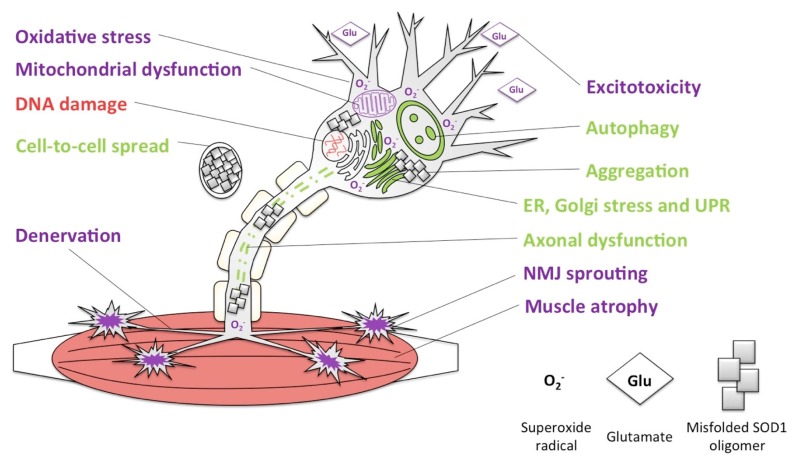
SOD1 loss-of-function and toxic gain-of-function cause many shared consequences. Red labels denote consequences from SOD1 loss of function (LOF); green labels are consequences of toxic gain of function (GOF); and purple labels show consequences seen in both cases. SOD1 toxic GOF and LOF result in a partial or total loss of oxidative function, leading to buildup of free radicals (e.g., O_2_^−^), oxidative stress, mitochondrial dysfunction, and may trigger apoptosis. Misfolded SOD1 acquires a toxic GOF that includes buildup of aggregates, misfolded protein spread, ER stress (endoplasmic reticulum stress) and Golgi trafficking disruption, unfolded protein response (UPR), autophagy and disruption of axonal trafficking of mitochondria. Both LOF and GOF lead to oxidative stress, mitochondrial dysfunction, sensitivity to excitotoxicity, neuromuscular junction (NMJ) sprouting, denervation and consequently muscle atrophy. The significant overlap in cellular consequences of LOF and GOF suggest that both mechanisms are at play in in ALS.

The role of SOD1 loss-of-function in ALS is a topic of exploration in the literature, though there are few studies we are aware of that assess the abundance of normally-folded SOD1 protein over the course of disease (Figure 8) [107]. Available SOD1 knockout mouse models show phenotypes to support this contention, although these were initially thought to develop normally with few overt signs of neurodegeneration [107,108]. It is now understood that SOD1 null mice are subject to increased oxidative stress and are more susceptible to neuron loss following axonal and ischemic brain injuries [108,109], changes in neuromuscular junction structure and function [110], muscle oxidative stress and associated atrophy, and progressive distal motor neuron degeneration associated with high oxidative stress [110,111,112,113]. Heterozygous mice also have reduced oxidative capacity [114]. Many but not all ALS patients show diminished SOD1 activity or increased oxidative stress, thus loss of function cannot fully account for disease. Nevertheless, based on our understanding of the roles of SOD1 in healthy neurons and the consequences of its absence in null and haploinsufficient models, reduction or complete loss of SOD1 function would further weaken a neuron’s ability to cope with stresses associated with misfolded protein accumulation [115,116]. Current therapeutic approaches that include reducing normally-folded SOD1 may thus have unwanted consequences.

**Figure 9:**
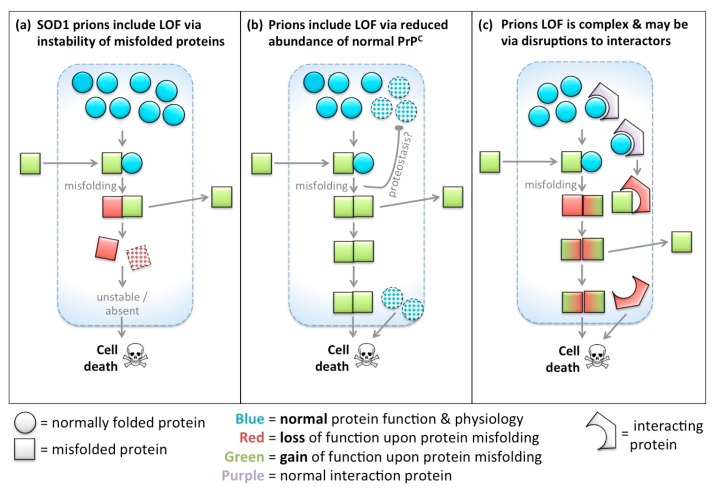
Loss or reduction in protein function appears to be required in prion-like disease. (**a**) Because SOD1 is unstable upon misfolding, some of its functions and abundance are reduced; thus in this instance prion-like induction of template-directed misfolding is the required gain of function, but reduced functions might drive ALS aetiology; (**b**) Similarly, normally folded prion protein (PrP^C^) is reduced in its abundance in at least some prion disease paradigms. Examples include in the preclinical phase of mouse models and in at least some key brain regions of patients with Fatal Familial Insomnia. Mechanisms of this PrP^C^ reduction are unknown but at least in the former they seem to include proteostatic degradation of PrP^C^. Reductions in PrP^C^ functions such as neuroprotection may directly contribute to disease aetiology; (**c**) Misfolding of proteins such as PrP^C^ and other prion-like proteins is expected to also impact upon the proteins with which they interact. If these interacting proteins are neuroprotective or required for neuron survival then their lost functions will contribute to aetiology. Also represented is gradations of both gain- and loss-of-function within the same misfolded protein, meant to acknowledge that it may be only some functions are partially lost upon misfolding though this certainly can also contribute to disease. ALS, Amyotrophic Lateral Sclerosis.

The considerably less intense phenotype of the SOD1 knockout mouse compared to the mutant SOD1 transgenic models shows that loss of function is not sufficient to kill motor neurons, but it may be a significant contributor. Many changes found in neurons with misfolded SOD1 overlap with changes in SOD1 knockout neurons, including increased oxidative stress, risk of excitotoxicity, neuromuscular junction changes, denervation and muscle atrophy. A significant part of this overlap can be explained by the reduced oxidative activity of all misfolded SOD1 proteins measured to date [107]. While it is not clear if the abundance of normally folded SOD1 decreases with disease course (as found with PrP^C^ in prion diseases, see below), certainly the propagated misfolding of SOD1 to conformers with reduced function could explain the LOF component in ALS pathology (Figure 9). Other processes in ALS including ER stress (endoplasmic reticulum stress), mitochondrial dysfunction, oxidative damage and apoptosis could conceivably result from either misfolded protein toxicity and aggregation, or SOD1 loss of function: the difficulty lies in determining the relative contribution of each toward pathologies. What is yet to be dissected is whether wildtype SOD1 is more likely to reduce aggregation and spread [99], or to become victim to conversion.

Some caveats might be considered regarding SOD1 being decreased in function during ALS, and thus regarding our assertion that SOD1 LOF is associated with ALS etiology. For example, SOD1 may not be reduced in all cell types or in all cell compartments. Furthermore the diversity of mutations that occur in ALS, and the involvement of disparate genetic lesions in other loci, suggests that ALS is likely not a single homogenous disease and SOD1 might behave with different kinetics in each instance. Further, it is likely that only some cell types are susceptible to SOD1 LOF-induced stress, i.e., some classes of neurons. ALS and frontotemporal dementia are understood to be on a continuum [117], and SOD1 null mice develop retinopathies similar to age-related macular degeneration (AMD) and glaucoma, presumably due to increased oxidative stress [118,119,120].

The prion diseases may have a similar under-appreciated LOF component. Both PrP^C^ and Shadoo (a prion family member and PrP^C^ interactor) decrease in abundance during aetiology of disease [121,122]. This decrease occurs during the pre-clinical phase of disease, prior to the appearance of disease symptoms, and this timing is consistent with reduced PrP^C^ function having an opportunity to impact upon aetiology.

Intriguingly, these alterations in PrP^C^ (and Shadoo) are not explained by changes at level of transcription, and cannot be accounted for by conversion to misfolded PrP^Sc^ [121,122]. This alludes to an active process of proteostasis.

The decrease in PrP^C^ is non-trivial to assess, as it requires conformationally-dependent immunoassays (CDI), and reagents that can faithfully discriminate PrP^C^ from PrP^Sc^ [121,122]. Thus information about the abundance and kinetics of normally folded species of prion-like disease mediators (e.g., Tau, SOD1) is not available during disease course. A further complexity is considering that decreases in normal protein abundance may be limited to certain brain regions, certain cell types, or may occur only in certain subtypes of disease or with certain strains.

Strikingly, detailed analysis of patients with an inherited human prion disease, Fatal Familial Insomnia, also reveals that PrP^C^ is significantly reduced in abundance at the end of disease course [123]. PrP^C^ reductions were only noted in the cerebellum, entorhinal cortex and thalamus [123], again speaking to the complexity of appreciating LOF amongst the complexity of in vivo systems. Fatal Familial Insomnia patients exhibit dramatic sleep disruptions, and these brain regions are known to be impactful on sleep [124,125]. This is speculatively suggestive that PrP^C^ reduction and general LOF in these regions participates in disease aetiology. Consistent with this notion, PrP^C^ LOF via knockout of *Prnp* in mice produces circadian rhythm defects [60].

Future works might consider whether the abundances of properly folded proteins are changing during disease course in other prion-like diseases. However this is no small undertaking: Even for prion diseases with their highly predictable clinical onset, it is only recently that careful CDI assays revealed the reductions in PrP^C^ early in the clinical phase [121,122]. Further, the reductions in abundance might be limited to only certain brain nuclei (as described above for Fatal Familial Insomnia patients), or even to cell types (e.g., neurons vs. glia) or sub-cellular compartments. Thus analysis of whole brain homogenates would typically mask changes in abundance occurring in smaller regions and compartments. Regardless an increased focus on disease-specific epitopes as therapeutic avenues may also provide the tools required to assess fundamental questions regarding differential abundance of protein confomers, e.g., via CDI.

## 7. Loss and Gain of Function in Amyloidosis: What News from Functional Amyloid?

**Figure 10:**
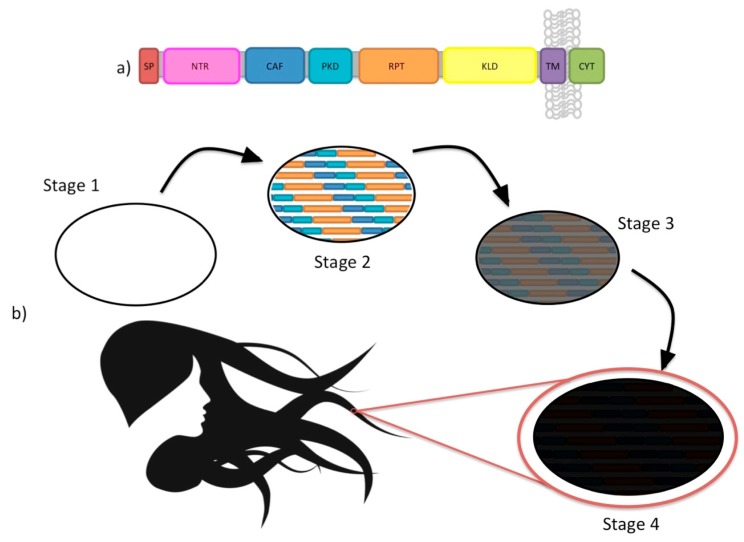
Premelanosome protein (PMEL) is an intriguing example of a vertebrate functional amyloid that, when processed, forms fibrils within melanosomes. PMEL in melanosomes sequesters the pigment melanin and is responsible for the pigment present in our eyes, hair, and skin. (**a**) A general schematic of the domains and regions of the PMEL protein: Signal Peptide (SP), N-terminal Region (NTR), Core Amyloid Fragment (CAF), Polycystic Kidney Domain (PKD), Repeat Region (RPT), Kringle-like Domain (KLD), Transmembrane Domain (TM), Cytoplasmic Domain (CYT); (**b**) The process of melanosome maturation and the integral function of PMEL in the sequestering of melanin pigment, which can be found in the skin, hair, and eyes. Stage 1: Also called the early endosome stage, this is the initial stage of a melanosome before PMEL fibrils are formed within the organelle. Stage 2: In the stage 2 melanosome, processed fragments of PMEL, i.e., CAF, PKD, and RPT, fibrilizes in the melanosome. Stage 3 and Stage 4: Melanin deposits onto the fibrils of PMEL giving the organelle its dark pigment.

Across vertebrates, a small collection of proteins are known to form functional amyloid as a required step in their normal physiological roles during health [126,127,128]. This includes premelanosome protein (PMEL), which assembles into a amyloid to perform as a key structural element critical to the deposition of pigment in melanosomes, the melanin-rich organelle in pigmented cells (Figure 10). Notably, other vertebrate proteins form amyloid to assist in their storage (e.g., somatostatin [129]) but as of yet these amyloids have not been found to possess a function unto themselves such as an enzymatic activity, or structural or physiological roles. Other functional amyloids play roles in signal transduction [130] and perhaps in stress granules [131]. Outside the vertebrates, especially amongst unicellular organisms, the list of functional amyloids identified to date is longer and more diverse but these are not considered further here.

Amongst the functional amyloids, PMEL appears to be a likely candidate to inform about loss and gain of function regarding amyloidosis. Though PMEL is widely studied as a biomarker in melanoma (being dysregulated during progression), most researchers seem to not consider that it might have a functional role (or lost role) during cancer biology. Indeed no mutations in PMEL have been reported to be associated with human disease at the time of writing. Intriguingly, though, disrupted PMEL produces phenotypes across disparate vertebrates; below we thus return to the farmyard to inform questions of proteostatic mechanisms and loss of function during amyloidosis.

PMEL is processed into fibrils that in the compartment of a melanosome, sequesters melanin, therefore, allowing for the presence of pigment in structures, such as hair, skin, and eyes [132]. The processing of PMEL is very similar to that of APP, which, when processed by a β-secretase, beta-site cleaving enzyme 1 (BACE1), and a γ-secretase, generates Aβ. PMEL protein, similarly, is cleaved by a β-secretase, i.e., BACE2 [133], and also a γ-secretase. Rather than being pathogenic akin to APP and Aβ, the proper processing of PMEL is required for normal phenotypes and healthy development [134,135] (Figure 10).

The phenotypes associated with PMEL naturally occur in many species and appear as either silver coloured coats of fur, mottled coats of fur, or a deficit of pigment deposition in the skin. Animals where PMEL has been determined to be the causative gene for cases of abnormal pigmentation include: horses, cattle, mice, dogs, zebrafish, and chickens [134,135,136,137,138,139,140,141]. An association with ocular phenotypes is also present in several species [136,139,140,141].

In order to explore the interaction of GOF and LOF in prion and prion-like diseases, we will use PMEL mutations in chickens as a case study supporting theories where both GOF and LOF of a protein contributes to a phenotype [135]. The chicken makes a remarkable case study for the effects of GOF and LOF because there are several phenotypes that are associated with mutations in the PMEL gene: *dominant white*, *smoky*, *dun*, *black langshan*, and *broiler*. The most interesting of these is the interplay between the phenotypes of dominant white and smoky.

**Figure 11:**
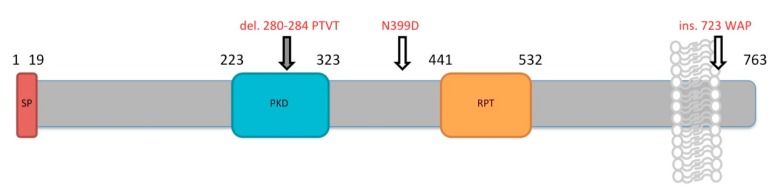
A schematic of chicken premelanosome protein (PMEL). A wild-type chicken has black, red, and orange plumage. The two mutations that cause the *dominant white* phenotype, i.e., plumage that is stark white, are N399D and a three amino acid insertion (residues tryptophan-alanine-proline, WAP) at amino acid 723. An additional deletion of four amino acids (residues proline-threonine-valine-threonine, PTVT), in the polycystic kidney domain (PKD), in addition to the two mutations found in the dominant white chicken, cause the smoky phenotype, i.e., plumage that is dark gray in colour.

The dominant white phenotype has two mutations in the PMEL gene. The first is a base change at amino acid 399, located between the polycystic kidney domain (PKD) and the repeat domain (RPT), from an asparagine to an aspartic acid. The second is an in-frame insertion at amino acid 723, located near or within the transmembrane domain, resulting in coding of three additional amino acids: tryptophan, alanine, and proline (Figure 11). It is accepted that the causative mutation is the three amino acid insertion near the transmembrane domain causing the loss of pigment due to PMEL. This mutation results in plumage that is devoid of colour, resulting in the dominant white phenotype [135].

The smoky phenotype is a sub-lineage of the dominant white phenotype, meaning that smoky chickens have the two mutations that are present in chickens with the dominant white phenotype, but also have one additional mutation which changes the white plumage, characteristic of the *dominant white* chicken, to the mottled gray colour characteristic of the smoky chicken phenotype. The additional mutation is an in-frame deletion resulting in the loss of the four amino acids, 280–284, in the PKD domain: proline, threonine, valine, and another threonine (Figure 11). The presence and effects of this mutation presents an interesting case study on the interplay between LOF and GOF to cause particular phenotypes, because the deletion of these four amino acids results in the regaining of function, but not complete recovery of function of the mutant *dominant white*, resulting in semi-pigmented plumage [135].

It has been proposed that the mutations found in dominant white chickens causes a change in PMEL which leads to the aberrant formation of amyloid fibrils, and to the complete lack of pigmentation [142]; this would imply that the *dominant white* mutation has both a GOF and LOF effect. The additional deletion found in the *smoky* lineage is thought to cause a null allele, and somehow result in diluted but not absent pigment [142], which is consistent with a similar dilution of pigment found in PMEL null mice [143]. However, the four amino acid deletion that separates the smoky lineage from the dominant white lineage is not naturally found in chickens of a wild-type background; therefore, the effects of the deletion alone are currently unknown [135].

Protective functions of PMEL amyloid may include mitigating oxidative stress. Intermediates of melanin synthesis are known to be oxidative. Intriguingly, these oxidative intermediates are noted to be similar in structure of amyloid binding dyes such as thioflavin T, might be bound by PMEL amyloids and thus neutralized [128,144,145].

In Sum, physiological PMEL as a functional amyloid provides an intriguing new context to LOF during aggregation of protein towards making amyloid. Two lessons can perhaps be derived: (i) Formation of amyloid can be dissociated from protein LOF—in certain cases the proteins aggregating towards amyloidosis retain their function; (ii) amyloid can be managed by healthy cells for a lifetime (though this is true for several amyloids, it is not clear if these are all special cases, i.e., some amyloids may be toxic). As a logical extension from these points, it is apparent that some proteins like PMEL can simultaneously: aggregate into amyloids; not lose their function; not be toxic to cells.

In this instance, then, it seems that LOF might be a requirement for amyloidosis to cause toxicity—however this remains as conjecture based on a correlated cluster of effects (or the absence of effects) that remains to be tested experimentally. Also, as noted above, it is not clear at this time whether this is a special class of amyloid that is distinct from the disease-related (and presumably toxic) amyloids such as Aβ, Huntingtin, α-Synuclein or PrP^Sc^.

## 8. Challenges of Disentangling GOF from LOF: Experiments Cannot Prove that GOF Is Sufficient

A key result from this synthesis is that gained functions are probably not sufficient in prion-like diseases. To challenge this conclusion, or to identify exceptions, one must be able to demonstrate sufficiency of gained function(s) to produce disease without impacting the functions that are potentially reduced, subverted or lost. This approach could be made more practical by instead asking if gained function(s) can at least induce some symptoms or processes associated with the disease course.

Unfortunately, the molecular events that define prion and prion-like disorders seem to preclude this type of analysis. The defining feature of these diseases is a gained function that the misfolded protein induces further misfolding/aggregation of physiological/healthy protein. Given that misfolding is accompanied by reduced and subverted function, in the prion-like diseases it is hard to conceive of an experimental test for protein GOF wherein LOF is not also occurring [7].

While many symptoms of the various prion-like diseases can be recapitulated when the prion-like protein is lost (e.g., Figure 4), we are aware of no example where a disease symptom can be demonstrated experimentally to be induced solely by GOF. GOF cannot be confidently ascribed as sufficient for disease etiology in any current prion or prion-like disease system, because it can never be fully disambiguated from LOF.

## 9. Interventions to Test If Loss of Function Plays a Role in Prion-Like Diseases

Separately, one may consider that versions of PrP^C^ can be imagined that would not participate in misfolding (not increase the acceleration of prion disease progression by participating in gain-of-function) but would have PrP^C^’s normal functions and could be supplied therapeutically, as we and others [7,146] recently suggested. For example rabbit PrP^C^ is known to not participate in prion biology [147,148]—it is not misfolded by prions and thus does not propagate disease, for example in transgenic mice. Can it block disease progression when mouse PrP^C^ is also present to template protein misfolding? Indeed such approaches appear promising, because heterologous hamster PrP^C^ can be used to treat scrapie infected mice, delay disease symptoms and reduce the amount of PrP^Sc^ formed [149].

While few tests are available to disentangle LOF from GOF, as reviewed above, one experimental approach is to ask if mutant (or misfolded) protein retains its normal physiological functions. We recently reviewed examples where this has been applied to inform ALS, Huntington, and Alzheimer Disease via delivery of mutant genes to rescue knockout animals [7]. Examples include rescuing the lethality of Huntingtin knockout mice using mutant disease-associated Huntingtin protein: The disease-associated variant retains some of its functions [150], at least prior to oligomerization. Also, including disease-associated mutations in human APP blunt its ability to recue APP knockdown in zebrafish [151,152]. We are not aware of this insightful approach being utilized in other instances, and this is especially notable for SOD1 and Tau, though further work on this approach seems warranted for each of the prion-like genes. Future works with these approaches might also ask if the timing of LOF and GOF affect the disease course. Conditional transgenes, for example those controlled by Cre recombinase, might enable addition (or subtraction) of wild type and mutant proteins at various stages of disease, to assess if LOF is required only early or late in disease (e.g., see schema in Figure 2 and Figure 9).

Indeed Cre-mediated excision of *PRNP* and reduction of PrP^C^ in neurons after prion inoculation has been demonstrated to reverse disease course [153]. En face, this represents a challenge to our hypothesis that loss of prion function contributes to disease progression. The loss of PrP^C^ can mediate two effects: (i) deceleration of disease progression by removing template for spreading and GOF; (ii) losing the normal functions of PrP^C^. These effects are imagined to contribute differentially in different cell types and in different phases of disease. Experiments removing PrP^C^ from neurons [153] show that LOF is not a large contributor in all cell types or in all phases of disease. It would be insightful to remove PrP^C^ during later phases of disease, and form other supporting non-neuronal cell types. Further, it is intriguing to note that the Cre-mediated loss of neuronal PrP^C^ did not blunt accumulation of misfolded PrP^Sc^, however PrP^Sc^ was reduced in the neurons themselves [153] (accumulations were in supporting cells); thus GOF of prion misfolding was also removed from the neurons during this PrP^C^ ablation, again demonstrating that GOF is required for prion disease progression. Disambiguating LOF from GOF in such scenarios remains challenging to accomplish experimentally.

A parallel approach is underway, compelling that the aggregation and toxicity of prions are separable properties. Therein self-templating but non-toxic strains of prions are encouraged to propagate in rodent models. These ‘anti-prions’ blunt the infectivity of prions when delivered as prophylactic treatments [154].

It would be intriguing to ask if PrP^C^ levels decrease during the production of these self-templating non-toxic prion strains. In the framework of our Hypotheses (see Section 3 and Figure 2) the existence of prions that are non-toxic despite being aggressive in producing prion aggregation argues that some prion strains have most every element of gained function without inducing toxicity. This is reminiscent of conclusions from the consideration of functional amyloids (Section 7) and consistent with the conclusion that GOF is necessary but not sufficient for disease etiology. It also urges a complementary question: if GOF in this system is not producing disease, which key functions of PrP^C^ (and its interactors) are lost during disease course—i.e., which functions of PrP^C^ are dispensable to a healthy brain? Perhaps no PrP^C^ functions are lost or reduced. One might begin by assessing abundance of PrP^C^: In the few instances where the question is carefully addressed, PrP^C^ levels are markedly reduced in prion disease (see Section 6). If this same proteostatic process is occurring vibrantly in the newly identified self-templating non-toxic prion strains, it might serve as a notable rebuttal to our hypotheses; minimally it would cause us to ask if there are some functions gained in disease-causing strains (leading to toxicity) that are not universal to prion aggregation. On the other hand, it might remain challenging to incisively address whether PrP^C^ functions are lost in any such system, especially considering the field’s diffuse understanding of what these many functions all entail (reviewed in Section 4).

## 10. Conclusions

We argue herein that it may be an unhelpful over-simplification to consider the misfolding of prions (and prion-like proteins) to be a GOF event that generates toxic elements. Indeed, we present support from diverse corners for our alternate hypothesis, that the GOF is required (i.e., it produces the critical element of spreading and template-directed misfolding) but the deregulation of physiology towards neurodegeneration may largely rest in downstream LOF events.

We use SOD1 as an informative case-study here, because it seems to best represent several elements: (i) SOD1 is unstable and reduced in function following misfolding; (ii) the normally folded functional SOD1 is important to normal cell function and inherently neuroprotective; (iii) misfolding of SOD1 leads to loss of its normal cellular functions. It is useful to re-emphasize here that elements of this list may be applied to all prion-like disorders: while the former element (i) may be less ubiquitous, the latter elements (ii) and (iii) of losing normal protein physiology and normal protein interactions that underpin neuroprotective functions appears common in prion-like systems. Therefore while GOF is required, as it is the definition of a disease being classified as prion-like, it is not difficult to imagine that LOF may be a required aspect (and in some instances even a dominant aspect) of the etiology in the prion-like diseases.

## Abbreviations

AD	Alzheimer Disease
ALS	Amyotrophic Lateral Sclerosis
Aβ	Amyloid-β
AβPP	Amyloid-β Precursor Protein
CWD	Chronic Wasting Disease
GOF	Gain of Function (following protein misfolding/aggregation)
LOF	Loss of Function (following protein misfolding/aggregation)
PrP^C^	Prion Protein, cellular form (normally folded form in healthy brains)
PrP^Sc^	Prion Protein, Scrapie form (misfolded form, disease-causing)
SOD1	Superoxide Dismutase 1
TDM	Template Directed Misfolding
